# Cooking methods are associated with inflammatory factors, renal function, and other hormones and nutritional biomarkers in older adults

**DOI:** 10.1038/s41598-022-19716-1

**Published:** 2022-10-01

**Authors:** Montserrat Rodríguez-Ayala, José Ramón Banegas, Rosario Ortolá, Manuel Gorostidi, Carolina Donat-Vargas, Fernando Rodríguez-Artalejo, Pilar Guallar-Castillón

**Affiliations:** 1 Department of Preventive Medicine and Public Health, School of Medicine, Universidad Autónoma de Madrid and CIBERESP (CIBER of Epidemiology and Public Health), 28029 Madrid, Spain; 2 Department of Microbiology and Parasitology, Hospital Universitario La Paz, 28046 Madrid, Spain; 3 Department of Nephrology, Hospital Universitario Central de Asturias, Red de Investigación Renal (RedinRen), 33011 Oviedo, Spain; 4 ISGlobal, Campus Mar, 08036 Barcelona, Spain; 5 Universitat Pompeu Fabra (UPF), 08002 Barcelona, Spain; 6 Unit of Cardiovascular and Nutritional Epidemiology, Institute of Environmental Medicine, Karolinska Institutet, SE-171 77 Stockholm, Sweden; 7 IMDEA-Food Institute, CEI UAM+CSIC, 28049 Madrid, Spain

**Keywords:** Nutrition, Geriatrics, Epidemiology, Biomarkers

## Abstract

Evidence of the role of cooking methods on inflammation and metabolic health is scarce due to the paucity of large-size studies. Our aim was to evaluate the association of cooking methods with inflammatory markers, renal function, and other hormones and nutritional biomarkers in a general population of older adults. In a cross sectional analysis with 2467 individuals aged ≥ 65, dietary and cooking information was collected using a validated face-to-face dietary history. Eight cooking methods were considered: raw, boiling, roasting, pan-frying, frying, toasting, sautéing, and stewing. Biomarkers were analyzed in a central laboratory following standard procedures. Marginal effects from generalized linear models were calculated and percentage differences (PD) of the multivariable-adjusted means of biomarkers between extreme sex-specific quintiles (Q) of cooking methods consumption were computed ([Q5 − Q1/Q1] × 100). Participants’ mean age was 71.6 years (53% women). Significant PD for the highest vs lowest quintile of raw food consumption was − 54.7% for high sensitivity-C reactive protein (hs-CRP), − 11.9% for neutrophils, − 11.9% for Growth Differentiation Factor-15, − 25.0% for Interleukin-6 (IL-6), − 12.3% for urinary albumin, and − 10.3% for uric acid. PD for boiling were − 17.8% for hs-CRP, − 12.4% for urinary albumin, and − 11.3% for thyroid-stimulating hormone. Concerning pan-frying, the PD was − 23.2% for hs-CRP, − 11.5% for IL-6, − 16.3% for urinary albumin and 10.9% for serum vitamin D. For frying, the PD was a 25.7% for hs-CRP, and − 12.6% for vitamin D. For toasting, corresponding figures were − 21.4% for hs-CRP, − 11.1% for IL-6 and 10.6% for vitamin D. For stewing, the PD was 13.3% for hs-CRP. Raw, boiling, pan-frying, and toasting were associated with healthy profiles as for inflammatory markers, renal function, thyroid hormones, and serum vitamin D. On the contrary, frying and, to a less extent, stewing showed unhealthier profiles. Cooking methods not including added fats where healthier than those with added fats heated at high temperatures or during longer periods of time.

## Introduction

Classical nutritional epidemiology has mostly studied the role of foods, food groups, nutrients and energy intake as well as dietary patterns on the prevention and management of chronic diseases^1,2^. However, food preparation, including cooking methods, have only recently received attention.

Indeed, the study of cooking methods has been very limited. One of the main reasons is the lack of data on cooking methods in large-sized studies, particularly when diet is collected using food frequency questionnaires. Besides, information on cooking was usually obtained in small studies focusing on changes in nutrient composition and physicochemical quality when a limited number of foods were subjected to different types of cooking^3,4^.

Frying is the most frequently studied cooking method in large populations, both in the United States and in Spain. In the United States, analyses from the Nurses' Health Study and the Health Professionals Follow-Up Study showed an association between fried food consumption and higher incident diabetes, cardiovascular disease and heart failure^5,6^. Similar results were obtained in a large sample of American veterans^7^. However, in Spain no association was found between the consumption of fried food and incident cardiovascular disease^8,9^. Other cooking methods have scarcely been investigated^10^.

Nevertheless, the study of cooking methods could provide interesting insights into the investigation of chronic diseases development given that many of these are related to changes in inflammatory, renal, and other nutritional biomarkers^11,12^. Thus, in a previous large-sized study, cooking patterns showed a relationship with inflammatory and cardio-metabolic health biomarkers^13^. More specifically, four cooking patterns were found: (a) The “Spanish traditional pattern”, characterized by boiling, sautéing, brining, and pan-frying, tended to be metabolically advantageous; (b) the “Health-conscious pattern”, based on battering, frying, and stewing, seemed to improve renal function; (c) the “Youth-style pattern”, with frequent consumption of soft drinks and distilled alcoholic drinks, and low consumption of raw food, was associated with metabolic benefits, except for a higher insulin and higher urinary albumin levels; and (d) the “Social business pattern”, rich in fermented alcoholic drinks, food cured with salt or smoked, and cured cheese, appeared to be detrimental for lipid profile, renal function and other metabolic biomarkers.

Therefore, the aim of the study was to evaluate for the first time in literature, the association of eight cooking methods with a wide array of inflammatory, renal, and other hormones and nutritional biomarkers in a population-based sample of older adults. In this population, these parameters have been little examined, and might reflect their metabolic and renal health profiles. Our initial hypothesis was that frying could be a detrimental form of cooking, while boiling, pan-frying, and sautéing could be beneficial cooking methods.

## Methods

### Study design and participants

The Seniors-ENRICA-2 study consists of 3273 individuals aged 65 years and older. Analyses were performed with baseline information of this cohort including those recruited from 2015 to 2017. Participants came from community-dwelling population of Community of Madrid (Spain) that included its capital, Madrid, and another four surrounding municipalities with large populations: Alcalá de Henares, Alcorcón, Getafe, and Torrejón de Ardoz. Participants were selected among those with a national health care card and following a sex- and district-stratified random sampling. Of note is that access to the health care system is universal in Spain.

Information was obtained in three stages, with similar procedures to the Seniors-ENRICA-1 study^14^. Sociodemographic and lifestyle characteristics, morbidity, and healthcare use were obtained by a telephone interview. Then, blood and urine sample collections along with a physical examination were performed in a home visit. Seven days later an interviewer performed a face-to-face dietary history.

From the initial sample of 3273 participants, a total of 806 were excluded: 483 (14.8%) without dietary information, 12 (0.4%) with implausible values for total energy intake (< 800 kcal/day or > 5.000 kcal/day in men; < 500 kcal/day or > 4.000 kcal/day in women), 20 (0.6%) lacking data on potential confounders, and 291 (8.9%) with missing information on laboratory biomarkers. Therefore, 2467 participants remained for main analyses; a nested subsample was used to analyze some biomarkers as described in Biomarkers section below.

All methods were carried out in accordance with relevant guidelines and regulations. All participants gave written informed consent. The Clinical Research Ethics Committee of La Paz University Hospital in Madrid provided ethics approval.

### Dietary information and cooking methods

Information on cooking methods was collected by trained and certified non-medical interviewers through a validated dietary history, DH-ENRICA. Information on habitual food consumption was collected considering all food consumed at least once every two weeks during the year prior to the interview. DH-ENRICA comprises 860 foods along with their cooking technique or preservation method. To estimate portion sizes, 127 sets of digitalized photos, household measurements and information on food proportion of typical ingredients from Spanish recipes were used^15^. Then, food consumption according to their cooking method was summed up in grams per day.

Eight cooking methods with a minimum mean consumption of 15 g/d were included in this analysis: raw (not cooked), boiling (cooking in aqueous medium with heat transmission by water), roasting (dry cooking transmitted by air or radiation), pan-frying (high temperature dry cooking using a pan and a minimum amount of oil), frying (high temperature cooking with food immersed in hot fats or oils; breaded, floured or battered food were also considered in this category), toasting (browning food using heat radiation), sautéing (brief medium to high heat cooking in a low-fat pan), and stewing (low heat cooking, usually simmered food in liquids for a long period of time). Mixed cooking methods (e.g. boiling + sautéing, frying + boiling, or sautéing + roasting) were not considered for this analysis since these are not frequent in Mediterranean diet^16,17^ and were barely consumed by participants in this study (less than 10 g per day).

### Biomarkers

At first home visit, 12-h fasting blood and urine samples were collected by nurses using standardized procedures. Inflammation markers were measured, including high-sensitivity C-reactive protein (hs-CRP), white blood cell count (WBC), neutrophils, lymphocytes, platelets, growth differentiation factor-15 (GDF-15) and interleukin-6 (IL-6). Renal function was assessed with serum creatinine, urinary albumin, serum uric acid, serum sodium, and serum potassium. Chronic Kidney Disease-Epidemiology Collaboration equation (CKD-EPI) was used to assess glomerular filtration rate based on serum creatinine levels^18^. Hormones and other nutritional biomarkers included thyroid-stimulating hormone (TSH), serum vitamin D, serum albumin, and total serum protein. A description of biomarkers measurement scales and procedures is provided in Supplementary Table 1.

Some measurements were only determined in a 1066 participants subsample, corresponding to those recruited from January 1st, 2017 to the end of the study. These biomarkers encompassed: hs-CRP, urinary albumin, serum uric acid, serum sodium, serum potassium, TSH, serum vitamin D, and serum albumin. All samples were centrally analyzed in core laboratory at La Paz University Hospital in Madrid.

### Potential confounders

Participants provided information on age, sex, educational level (primary or less, secondary, university), smoking status (current, former or never smoker). Alcohol consumption was assessed using the dietary history. A specific question was answered to ascertain former-drinker status. Recreational physical activity and household physical activity was evaluated with the EPIC short physical activity questionnaire with 17 items. Each of them was multiplied by their respective energy expenditure rate in metabolic equivalent of task—METs·hour/week^19^ and total energy expenditure was calculated by summing up all physical activities. Number of television hours was used as sedentary lifestyle indicator. To assess morbidity, the number of chronic diseases and the number of medications prescribed (checked against packaging) were considered. To take into account diet quality and its influence in the results, models were also adjusted for the intake of very long chain omega-3 fatty acids (eicosapentaenoic acid—EPA, and docosahexaenoic acid—DHA), and for total fiber consumption.

### Statistical analysis

For each cooking method, analyses were performed in grams per kilogram (g/kg) of body weight to take into account body size. Participants were classified into sex-specific quintiles for each cooking method. Data on biomarkers were log-transformed in order to achieve normality. Marginal effects from generalized linear model (GLM) were obtained. The regression models were adjusted for sex, age, energy intake, educational level, smoking, alcohol consumption, former-drinker status, recreational physical activity in METs·hours/week, household physical activity in METs·hours/week, hours of television hours, number of chronic diseases, number of prescribed medications, intake of very long chain omega-3 fatty acids (eicosapentaenoic acid—EPA, and docosahexaenoic acid—DHA), and total fiber consumption. Geometric means were estimated, and percentage differences (PD) were calculated as [(5th quintile–1st quintile)/1st quintile] × 100. In this study, a PD greater than 10% was deemed clinically relevant. A similar cut-off point has been considered clinically relevant in other scenarios (such as in hypertension) when using nutritional recommendations for medical chronic conditions^20^. To calculate *p* for linear trend, quintiles of consumption were modelled as a continuous variable.

All analyses were performed with Stata, version 15.0 (StataCorp, College Station, Texas, USA), setting the statistical significance at *p* < 0.05.

## Results

The main analyses were performed with 2467 participants with a mean age of 71.6 (± 4.4) years, and 53.0% were women. Those collected after January 1st, 2017 (subsample, n = 1066) had a mean age of 70.8 (± 3.9) years, and 49% were women. Baseline characteristics of main sample according to quintiles of cooking methods are shown in Table 1. Significant differences were found when comparing extreme quintiles of consumption for each cooking method. In general, those in the highest quintile of consumption of raw and pan-frying had the healthiest lifestyle profile. However, those in the highest quintile of consumption of roasting, frying, and stewing showed a more harmful lifestyle profile.

**Table 1:** Baseline characteristics of participants in extreme quintiles of cooking methods. The Seniors-ENRICA 2 Study (N = 2467).

	Raw		Boiling		Roasting		Pan-frying		Frying		Toasting		Sautéing		Stewing	
	Q1	Q5	Q1	Q5	Q1	Q5	Q1	Q5	Q1	Q5	Q1	Q5	Q1	Q5	Q1	Q5
Age, mean, y	71.7	71.6	71.6	71.8	72.3	70.4^‡^	72.3	71.1^‡^	71.3	72.0*	71.7	71.6	71.9	71.6*	71.6	71.3
Women, (%)	53.0	53.1	53.0	53.1	53.0	53.1	53.0	53.1	53.0	53.1	46.3	53.1^‡^	53.0	53.1	53.0	53.1
**Education, (%)**																
Primary or less	64.8	58.7	55.9	64.2†	54.7	67.9^‡^	66.6	62.0	53.2	73.6^‡^	69.0	55.1^‡^	58.7	60.8†	59.7	64.0*
Secondary	17.8	19.5	20.6	19.5	20.9	18.3^‡^	16.4	19.1	21.7	13.6	17.2	20.1	17.4	20.7	17.2	17.9
University	17.4	21.8	23.5	16.3	24.5	13.8^‡^	17.0	18.9	25.1	12.8	13.7	24.8	23.9	18.5	23.1	18.1
**Smoking status, (%)**																
Current	12.5	6.71*	10.7	8.13†	7.5	11.6†	11.9	7.3	7.50	12.4^‡^	13.1	5.3†	6.88	10.8	9.31	9.55
Former	38.9	39.2	39.3	34.6	43.7	32.3	37.7	41.5	43.3	31.7	37.4	40.0	41.5	38.2	39.3	39.0
Never	48.6	54.1	50.0	57.3	48.8	56.1	50.4	51.2	49.2	55.9	49.5	54.7	51.6	51.0	51.4	51.4
Alcohol consumption, median, g/d	3.17	1.96	3.7	0.9^‡^	2.2	1.1^‡^	1.9	1.2	1.7	2.9*	2.7	1.8	3.5	1.8*	2.1	2.6
Former-drinker status, (%)	10.1	11.0	7.3	13.8†	10.3	11.6	10.9	11.0	12.6	10.0	10.3	10.6	8.10	8.94*	11.3	10.2
RPA^1^, median, METs·h/week	19.5	25.5^‡^	22.5	25.0	23.8	24.9	21.5	25.0^†^	24.6	25.5	22.5	25.0†	21.5	25*	21.5	25.0*
HPA^2^, median, METs·h/week	35.0	35.0	33.5	35.0	31.5	35.0*	35.0	35.0	34.0	36.8†	35.0	35.0	31.0	35.0*	35.0	35.0
Hours of television, median	23.9	20.3^‡^	22.9	21.7	21.4	22.4	23.2	21.3	21.8	22.3	22.8	20.8*	21.9	21.4	22.3	22.4
No. chronic diseases, median	1.0	1.0	1.0	1.0	1.0	1.0	1.0	1.0	1.0	1.0	1.0	1.0	1.0	1.0	1.0	1.0
No. medications, median	3.0	2.0^‡^	3.0	3.0	3.0	3.0	3.0	3.0	3.0	3.0	3.0	2.0^‡^	3.0	3.0	3.0	3.0
**Dietary variables**																
Energy intake, mean, kcal/d	2364.0	2385.9*	2249.6	2507.6^‡^	2276.0	2446.9^‡^	2438.0	2292.8^‡^	2213.7	2526.7^‡^	2377.5	2395.3*	2325.9	2405.0	2270.3	2439.7^‡^
Omega-3, median, g/d	0.486	0.595^‡^	0.529	0.533	0.545	0.527	0.386	0.779^‡^	0.460	0.683^‡^	0.512	0.546	0.519	0.547	0.539	0.612
Fiber, mean, g/d	25.8	35.3^‡^	26.7	34.9^‡^	30.1	30.9	30.1	30.6	30.0	30.8	28.9	32.1^‡^	31.2	30.2	29.6	30.9†

Q1 = Quintile 1 (lowest); Q5 = Quintile 5 (highest). Kruskal–Wallis test was used for comparisons between continuous variables and Pearson's Chi-square for categorical variables.

^1^RPA: recreational physical activity.

^2^HPA: household physical activity.

*p < 0.05; ^†^*p* < 0.01; ^‡^*p* < 0.001.

PD of inflammatory factors, renal function, and other hormone and nutritional biomarkers are provided in Table 2. A mean of 470 (± 208) g/d of raw food was consumed. PD for raw food consumption was − 54.7% for hs-CRP, − 11.9% for neutrophils, − 11.9% for GDF-15, − 25.0% for IL-6, − 12.3% for urinary albumin, and − 10.3% for uric acid. Considering boiling, a mean of 277 (± 119) g/d was consumed. PD for boiling was − 17.8% for hs-CRP, − 12.4% for urinary albumin, and − 11.3% for TSH. Concerning roasting, a mean of 156 (± 68) g/d was consumed. No relevant PD were found for roasting, although a trend towards lower urinary albumin and uric acid was observed. A mean of 63 (± 47) g/d of pan-frying food was consumed. PD for pan-frying were − 23.2% for hs-CRP, − 11.5% for IL-6, − 16.3% for urinary albumin, and showed an increase of 10.9% for serum vitamin D. A mean of 42 (± 33) g/d of frying food was consumed. PD for frying were 25.7 for hs-CPR and − 12.6 for vitamin D. Observing toasting, 42 (± 40) g/d was consumed. PD for toasting were − 21.4% for hs-CRP, and − 11.1% for IL-6. Also, a PD of 10.6% in serum vitamin D was observed. Regarding sautéing, a mean of 22 (± 21) g/d was consumed. No differences were observed between extreme quintiles for sautéing consumption. A mean of 19 (± 21) g/d of stewing food was consumed. PD for stewing was of 13.3% for hs-CRP. Adjusted means (95% confidence interval), percentage difference, and p for linear trend across quintiles of cooking methods consumption are presented in Supplementary Tables 2–9.

**Table 2:** Percentage differences (PD) of inflammatory factors, renal function, and other hormones and nutritional biomarkers among cooking methods.

Outcome variables	Cooking methods															
	Raw		Boiling		Roasting		Pan-frying		Frying		Toasting		Sautéing		Stewing	
	PD	*p*	PD	*p*	PD	*p*	PD	*p*	PD	*p*	PD	*p*	PD	*p*	PD	*p*
**Inflammatory markers**																
hs-CRP*	− 54.7	< 0.001	− 17.8	0.045	− 5.2	0.302	− 23.2	0.103	25.7	0.217	− 21.4	0.047	− 1.0	0.483	13.3	0.817
WBC	− 9.1	0.001	− 3.9	0.144	− 0.7	0.576	− 1.4	0.549	3.0	0.406	− 5.7	0.264	1.2	0.958	− 3.2	0.034
Neutrophils	− 11.9	0.002	− 5.9	0.045	− 1.5	0.181	− 3.0	0.831	4.1	0.185	− 7.3	0.253	0.0	0.316	− 2.1	0.273
Lymphocytes	− 2.9	0.08	− 1.8	0.766	0.0	0.483	1.8	0.363	1.2	0.955	− 2.3	0.985	3.6	0.079	− 4.5	0.021
Platelets	− 5.5	0.025	− 0.7	0.29	1.2	0.087	− 2.1	0.384	1.7	0.488	− 1.1	0.637	− 1.2	0.294	0.3	0.788
GDF-15	− 11.9	0.028	− 0.3	0.42	2.6	0.924	− 6.8	0.742	5.3	0.008	− 8.0	0.857	− 5.7	0.041	− 4.3	0.688
IL-6	− 25.0	0.001	− 4.0	0.139	8.3	0.800	− 11.5	0.404	4.2	0.978	− 11.1	0.009	0.0	0.851	4.0	0.777
**Renal function**																
Serum creatinine	− 2.5	0.075	− 2.5	0.317	0.9	0.355	− 3.8	0.201	1.3	0.317	− 1.8	0.782	− 3.4	0.004	− 1.3	0.976
CKD-EPI	2.1	0.044	0.8	0.213	0.1	0.134	2.3	0.794	− 1.7	0.197	1.5	0.636	2.5	0.020	1.7	0.243
Urinary albumin*	− 12.3	0.359	− 12.4	0.155	− 9.2	0.034	− 16.3	0.904	0.9	0.602	− 4.7	0.841	− 4.6	0.376	0.0	0.880
Serum uric acid*	− 10.3	< 0.001	− 9.2	< 0.001	− 8.6	0.003	− 4.2	0.204	− 2.7	0.159	− 3.8	0.253	− 4.7	0.003	1.0	0.035
Serum sodium*	0.2	0.008	− 0.1	0.357	0.1	0.931	0.0	0.951	0.0	0.438	0.1	0.354	0.1	0.074	0.0	0.558
Serum potassium*	− 2.9	< 0.001	3.7	0.003	2.5	0.016	− 0.4	0.316	3.9	0.001	0.2	0.726	− 1.0	0.239	0.5	0.715
**Hormones and nutritional biomarkers**																
TSH*	9.6	0.146	− 11.3	0.118	8.2	0.479	− 7.3	0.473	5.6	0.094	− 1.8	0.611	3.7	0.982	− 3.5	0.320
Vitamin D*	8.9	0.282	7.2	0.007	− 2.7	0.438	10.9	0.033	− 12.6	0.065	10.6	0.045	3.5	0.202	4.5	0.252
Serum albumin*	1.4	0.118	− 1.2	0.005	− 0.2	0.267	0.0	0.376	0.2	0.286	− 0.2	0.964	0.0	0.485	− 0.5	0.937
Serum total protein	0.3	0.934	0.3	0.768	− 0.6	0.233	− 0.3	0.938	− 0.7	0.022	− 0.1	0.718	0.1	0.121	0.1	0.690

PD: percentage difference; measured as a difference between quintile 5 and quintile 1 divided by quintile 1. Hs-CRP: high sensitivity-C reactive protein; WBC: white blood cells; GDF-15: Growth differentiation factor 15; IL-6: Interleukin 6; CKD-EPI: Chronic Kidney Disease-Epidemiology Collaboration equation; TSH: Thyroid-stimulating hormone.

*Calculated in the nested subsample (n = 1066).

*p*: *p* linear for trend of outcome variables when quintiles of consumption were used as a continuous variable in the model adjusted for all possible confounding variables (sex, age (continuous), energy intake (continuous), educational level (primary or less, secondary, university), smoking status (former, current, never), alcohol consumption (continuous), former-drinker status (yes, no), recreational physical activity in METs·hours/week (continuous), household physical activity in METs·hours/week (continuous), hours of television (continuous), number of chronic diseases (continuous), number of prescribed medications (continuous), very long chain omega-3 fatty acids (continuous), and fiber (continuous) consumption.

## Discussion

In this large population-based study on cooking methods, raw, boiling, pan-frying, and toasting showed beneficial profiles for inflammatory markers, while frying and stewing showed detrimental inflammatory profiles. Raw, boiling, and pan-frying showed beneficial differences in renal biomarkers; no other cooking methods influenced renal function. Boiling was associated with lower TSH levels. Pan-frying and toasting were associated with a higher serum vitamin D, while frying was associated with a lower level of this vitamin. Therefore, raw, boiling, pan-frying, and toasting are associated with healthy profiles. However, frying and, to a less extent, stewing showed unhealthier profiles. Cooking methods that included no added fats where healthier than those including added fats heated at high temperatures or during longer periods of time.

Our results, obtained in a sample of older adults, are an example of novel exposures influence that have hardly been studied in the past and that may also be used as a healthy ageing strategy^21^. Thus, although some cooking methods are considered healthy, and in many cases older adults have gradually adopted them, comprehensive information that provides guidance on food preparation has never been provided in literature before.

Several mechanisms could alter food while it is being processed, including thermal procedures and the addition of certain components such as fats. For instance, mechanical processing or soaking could also modify nutrient bioavailability^22^. Then, there is a wide range of processes involved: from liquid evaporation and its substitution with fats^23,24^, variations on antioxidant activity^25,26^, formation of advanced glycation end products^27,28^, to altered glycemic and insulinemic responses^29,30^. In addition, some specific cooking methods are considered harmful. During deep-frying, polycyclic aromatic hydrocarbons and heterocyclic amines are produced, which are genotoxic agents, and have been associated with cancer incidence^31^. Also, saturated fatty acids formation could increase during frying^32^ that, depending on its food source, are related varied biological effects.

Our findings support that, among the elderly, raw food consumption is associated with beneficial marker profiles (such as lower levels of hs-CRP, neutrophils, GDF-15, IL-6, urinary albumin, and serum uric acid). Two cross-sectional studies assessed the consumption of raw food-only diets. The first concluded that such a diet was associated with weight reduction, but also with underweight^33^. In the second one, an exclusive raw food diet was associated with lower LDL-cholesterol and HDL-cholesterol levels, in addition to vitamin B-12 deficiency^34^. Hence, along with general recommendations for a healthy diet (quality food choices and adequate daily consumption), it seems very reasonable to favor raw food consumption for this age group.

Regarding inflammatory markers, raw, boiling, and toasting food consumption were associated with lower hs-CRP levels. A possible explanation could rely on the fact that these cooking methods usually do not imply the addition of unhealthy fat sources^35^. In an analysis of the National Health and Nutrition Examination Survey (NHANES) conducted with 4900 adults, it was observed that saturated fat consumption was modestly associated with elevated hs-CRP^36^. In addition, raw food has high fiber and antioxidants contents, which are also associated with lower levels of hs-CRP^37^. On the contrary, frying and stewing were associated with higher hs-CRP levels in this study. The addition of harmful fats or a longer cooking time might be associated with higher levels of this inflammatory marker. Regarding this latter, recent studies have found that a longer cooking time—such in stewing, increases fat content of food^38,39^ that could lead to deleterious effects on health^40^.

Concerning other inflammatory markers, the association of raw food consumption (mainly fruit and vegetables) with lower counts of white blood cells and neutrophils was also examined in other studies. An observational study with 51 vegan participants found a reduction in CD4, CD8 and natural-killer cells^41^. Likewise, Mediterranean diet adherence, rich in raw food, was associated with lower leukocyte count levels^42^. This is important since neutrophils regulate inflammatory function and it has been suggested that lower neutrophil levels are associated with lower atherothrombotic processes and cardiovascular diseases^43,44^.

In our study, raw food consumption was also associated with lower GDF-15 levels. Among older adults, the importance of GDF-15 relies on its role as biomarker for cardiac fibrosis^45^ as well as a marker for chronic disease burden^46^. An essential cofactor of this biomarker is selenium—mostly found in raw consumed food^46^, and it has been described an inverse relationship between them^47^. On the other hand, although cooking methods were not evaluated, a 6-month clinical trial found no association between GDF-15 levels and plant-based or protein-based diets^48^.

Raw and toasting food consumption showed decreasing differences in IL-6 levels. These cooking methods involve dressing oils and hidden fats from fruits, nuts and seeds (usually consumed toasted)^49^ which in turn promote carotenoid absorption. Carotenoid levels are inversely related to serum IL-6 due to modifications in systemic inflammatory responses^50,51^. Similarly, in a cohort with 3075 older adults an association was found between lower IL-6 levels and a dietary pattern that included high consumption of fruit, vegetables, whole grains, and non-fried poultry^52^. Lower levels of IL-6 might be beneficial as an inverse relationship with coronary heart disease has been observed^53^.

Concerning renal function, there was a lower mean urinary albumin with raw, boiling, and pan-frying consumption in this population. A high renal excretion of albumin could reflect greater animal protein and fat intake, and, generally, these cooking methods do not involve the addition of fats. Therefore, these could be beneficial for both overweight and high blood pressure prevention^54^, and might also be recommended in patients with hypertension or decline in renal function. On the other hand, lower uric acid levels occurred with raw food consumption. This is in line with the literature as a lower purine content in raw food could be responsible for a lower uric acid formation^55,56^.

In relation to hormones and other nutritional biomarkers, our results suggest that boiling is associated with lower TSH levels and it could potentially prevent subclinical hypothyroidism. Though, further research is still needed. A study demonstrated that boiled cauliflower had lower levels of progoitrin, a precursor of goitrin^57^. However, other boiled cyanogenic plants (cabbage, radish, cassava) have shown high anti-thyroid peroxidase levels that could exert opposite effects^58^. Additionally, beneficial PD in serum vitamin D was observed with pan-frying and toasting. Conversely, the exposure to high temperatures has been shown to cause adverse effects in vitamin D3 content in food, but knowledge regarding this matter is limited, and the stability of vitamin D3 in added vegetable oils during cooking has not yet been determined^59,60^. Of note is that for pan-frying and toasting, food is exposed to heat for shorter periods of time.

The main strength of this study is the use of a dietary history that allowed to collect detailed information on food consumption and cooking methods. Its validity and reproducibility has been shown in Spanish population. Standardized procedures have been followed during analytical processing of biological samples in a central laboratory. Likewise, several confounding factors were taken into account (including socio-demographic determinants and lifestyle), although some degree of residual confounding cannot be ruled out.

This study has several limitations that have to be considered. First, since this study has a cross-sectional design, the results cannot establish causality. Second, as when a specific food is studied, it is not possible to distinguish the effect of cooking from the consumption of the food itself. Similarly, we cannot distinguish the effect of a specific cooking method from the effect of added fats when occurring together. Finally, because of administrative reasons, some measurements were only conducted in the subsample recruited from January, 2017 to the end of the study resulting in a reduction in sample size, but without deriving in selection bias.

## Conclusions

This study suggests that among older Spanish adults, some cooking methods, especially, raw, boiling, pan-frying, and toasting, are associated with positive differences in several inflammatory markers, renal function, thyroid hormones, and serum vitamin D. Therefore, these findings add information to current knowledge on the possible impact of cooking methods on health. Further studies are needed to establish causality and to fully understand the mechanisms underlying the relationships found.

## Supplementary Information

Supplementary Information.

## Data Availability

The datasets used and/or analyzed during the current study are available from the corresponding author on reasonable request.
