# Supplementary material for: Cooking methods are associated with inflammatory factors, renal function, and other hormones and nutritional biomarkers in older adults
Source: Sci Rep. 2022 Oct 1;12:16483. doi: 10.1038/s41598-022-19716-1 (PMC9526743; doi:10.1038/s41598-022-19716-1)
Supplement: Supplementary file 1 — Supplementary Information. [file 41598_2022_19716_MOESM1_ESM.docx]

**Manuscript: Cooking methods are associated with inflammatory factors, renal function, and other hormones and nutritional biomarkers in older adults**

# Montserrat Rodríguez-Ayala, José Ramón Banegas, Rosario Ortolá, Manuel Gorostidi, Carolina Donat-Vargas, Fernando Rodríguez-Artalejo and Pilar Guallar-Castillón

# Supplementary Tables

**Supplementary Table S1.** Units and techniques used for biomarkers measurement.

| **Biomarkers** | | **Units** | **Technique** |
| --- | --- | --- | --- |
| **Inflammatory markers** | |  |  |
|  | **Hs-CRP** | mg/L | Immunoturbidimetry technique using Atellica Solution® (Siemens Healthineers) |
|  | **WBC** | x 10^3^/µL | Flow cytometry. Advia 2120® (Siemens Healthineers) |
|  | **Neutrophils** | x 10^3^/µL | Flow cytometry. Advia 2120® (Siemens Healthineers) |
|  | **Lymphocytes** | x 10^3^/µL | Flow cytometry. Advia 2120® (Siemens Healthineers) |
|  | **Platelets** | x 10^3^/µL | Flow cytometry. Advia 2120® (Siemens Healthineers) |
|  | **GDF-15** | pg/mL | Chemiluminescent immunoassay using Cobas 6000 (Roche Diagnostics) |
|  | **IL-6** | pg/mL | Chemiluminescent immunoassay using Cobas 6000 (Roche Diagnostics) |
| **Renal function** | |  |  |
|  | **Serum creatinine** | mg/dl | Enzymatic method, colorimetric. Atellica Solution® (Siemens Healthineers) |
|  | **CKD-EPI** | mL/min/1.73m^2^ | Glomerular filtration rate calculated with CKD-EPI equation (2009). |
|  | **Urinary albumin** | mg/dl | Immunoturbidimetry technique using Atellica Solution® (Siemens Healthineers) |
|  | **Serum uric acid** | mg/dl | Spectophotometry with enzymatic method (uricase/peroxidase). Atellica Solution® (Siemens Healthineers) |
|  | **Serum sodium** | mEq/L | Indirect potentiometry. Atellica Solution® (Siemens Healthineers) |
|  | **Serum potassium** | mEq/L | Indirect potentiometry. Atellica Solution® (Siemens Healthineers) |
| **Hormones and nutritional biomarkers** | |  |  |
|  | **TSH** | μUI/mL | Chemiluminescent immunoassay using Atellica Solution® (Siemens Healthineers) |
|  | **Serum vitamin D** | ng/ml | Chemiluminescent immunoassay using Atellica Solution® Siemens Healthineers) |
|  | **Serum albumin** | g/dL | Spectophotometry with bromocresol green (BCG) with Atellica Solution® (Siemens Healthineers) |
|  | **Total serum protein** | g/dL | Direct colorimetric Biuret method, end-point reaction using Atellica Solution® (Siemens Healthineers) |

hs-CRP: high sensitivity C-reactive protein; WBC: white blood cells; GDF-15: Growth differentiation factor 15; IL-6: Interleukin 6; CKD-EPI: Chronic Kidney Disease- Epidemiology Collaboration equation; TSH: Thyroid-stimulating hormone.

**Supplementary Table S2.** Adjusted means (95% confidence interval), percentage difference, and *p* for linear trend across quintiles of raw food consumption.

|  | **Raw food consumption** | | | | |  | | |
| --- | --- | --- | --- | --- | --- | --- | --- | --- |
|  | **Q1** | **Q2** | **Q3** | **Q4** | **Q5** | **PD** | | ***p***^†^ |
| **Inflammatory markers** | |  |  |  |  |  |  | |
| **hs-CRP (mg/L)** | 1.79 (1.72-1.85) | 1.27 (1.22-1.31) | 1.31 (1.27-1.35) | 1.04 (1.01-1.09) | 0.81 (0.78-0.83) | -54.7 | <0.001 | |
| **WBC (10^3^/µ L)** | 6.07 (6.03-6.12) | 5.78 (5.74-5.83) | 5.76 (5.72-5.80) | 5.69 (5.65-5.73) | 5.52 (5.48-5.56) | -9.1 | 0.001 | |
| **Neutrophils (10^3^/µ L)** | 3.54 (3.51-3.57) | 3.27 (3.24-3.30) | 3.23 (3.20-3.26) | 3.26 (3.23-3.29) | 3.12 (3.09-3.15) | -11.9 | 0.002 | |
| **Lymphocytes (10^3^/µ L)** | 1.71 (1.70-1.72) | 1.73 (1.72-1.73) | 1.73 (1.72-1.74) | 1.67 (1.66-1.67) | 1.66 (1.65-1.67) | -2.9 | 0.080 | |
| **Platelets (10^3^/µ L)** | 236 (235-238) | 230 (228-231) | 229 (227-230) | 223 (222-225) | 223 (222-225) | -5.5 | 0.025 | |
| **GDF-15 (pg/mL)** | 1333 (1304-1361) | 1295 (1267-1323) | 1212 (1188-1237) | 1234 (1208-1259) | 1174 (1151-1197) | -11.9 | 0.028 | |
| **IL-6 (pg/mL)** | 2.8 (2.7-2.8) | 2.6 (2.6-2.6) | 2.5 (2.5-2.6) | 2.3 (2.3-2.63) | 2.1 (2.1-2.2) | -25.0 | 0.001 | |
| **Renal function** | |  |  |  |  |  |  | |
| **Serum creatinine (mg/dL)** | 0.80 (0.79-0.81) | 0.80 (0.79-0.81) | 0.78 (0.77-0.79) | 0.78 (0.77-0.79) | 0.78 (0.77-0.79) | -2.5 | | 0.075 |
| **CKD-EPI (mL/min/1.73 m^2^)** | 82.4 (81.7-83.2) | 83.0 (82.3-83.8) | 84.1 (83.3-84.9) | 84.1 (83.3-84.8) | 84.1 (83.3-84.8) | 2.1 | | 0.044 |
| **Urinary albumin** | 7.3 (7.0-7.5) | 7.5 (7.2- 7.7) | 6.1 (5.9-6.3) | 6.4 (6.2- 6.7) | 6.4 (6.2-6.6) | -12.3 | | 0.359 |
| **Serum uric acid (mg/dL)** | 5.44 (5.37-5.52) | 5.29 (5.21-5.37) | 5.25 (5.18-5.32) | 5.25 (5.18-5.32) | 4.88 (4.82-4.94) | -10.3 | | <0.001 |
| **Blood sodium (mEq/L)** | 140.5 (140.4- 141.5) | 14.5 (140.4-14.5) | 140.5 (140.5-140.5) | 140.6 (140.6-140.7) | 140.8 (140.8-140.9) | 0.2 | | 0.008 |
| **Blood potassium (mEq/L)** | 4.55 (4.54-4.57) | 4.48 (4.47-4.49) | 4.52 (4.51-4.54) | 4.42 (4.41-4.44) | 4.42 (4.40-4.43) | -2.9 | | <0.001 |
| **Hormones and nutritional biomarkers** | |  |  |  |  |  |  | |
| **TSH (μUI/mL)** | 1.57 (1.55-1.58) | 1.64 (1.63-1.66) | 1.72 (1.71-1.74) | 1.76 (1.74-1.78) | 1.72 (1.70-1.74) | 9.6 | 0.146 | |
| **Serum vitamin D (mg/dL)** | 16.8 (16.6-17.1) | 17.0 (16.7-18.1) | 17.9 (17.6-18.1) | 17.9 (17.6-18.1) | 18.3 (18.0-18.5) | 8.9 | 0.282 | |
| **Serum albumin (g/dL)** | 4.26 (4.26-4.27) | 4.32 (4.31-4.32) | 4.28 (4.27-4.28) | 4.30 (4.29-4.30) | 4.32 (4.31-4.32) | 1.4 | 0.118 | |
| **Total serum protein (g/dL)** | 7.04 (7.04-7.05) | 7.04 (7.03-7.05) | 7.02 (7.01-7.03) | 7.02 (7.01-7.03) | 7.06 (7.06-7.07) | 0.3 | 0.934 | |

hs-CRP: high sensitivity-C reactive protein; WBC: white blood cells; GDF-15: Growth differentiation factor 15; IL-6: Interleukin 6; CKD-EPI: Chronic Kidney Disease-Epidemiology Collaboration equation; TSH: Thyroid-stimulating hormone.

All analyses were adjusted for sex, age (continuous), energy intake (continuous), educational level (primary or less, secondary, university), smoking status (former, current, never), alcohol consumption (continuous), former-drinker status (yes, no), recreational physical activity in METs·hours/week (continuous), household physical activity in METs·hours/week (continuous), hours of television (continuous), number of chronic diseases (continuous), number of prescribed medications (continuous), very long chain omega-3 fatty acids (continuous), and fiber consumption (continuous).

^†^ *p* linear for trend of outcome variables when quintiles of consumption were used as a continuous variable.

**Supplementary Table S3.** Adjusted means (95% confidence interval), percentage difference, and *p* for linear trend across quintiles of boiling food consumption.

|  | **Boiling food consumption** | | | | |  | | |
| --- | --- | --- | --- | --- | --- | --- | --- | --- |
|  | **Q1** | **Q2** | **Q3** | **Q4** | **Q5** | **PD** | | ***p***^†^ |
| **Inflammatory markers** | |  |  |  |  |  |  | |
| **hs-CRP (mg/L)** | 1.35 (1.29-1.41) | 1.25(1.20-1.29) | 1.14 (1.09-1.19) | 1.10 (1.05-1.15) | 1.11 (1.06-1.15) | -17.8 | 0.045 | |
| **WBC (10^3^/µ L)** | 5.90 (5.86-5.95) | 5.66 (5.62-5.70) | 5.87 (5.83-5.91) | 5.72 (5.68-5.76) | 5.67 (5.63-5.72) | -3.9 | 0.144 | |
| **Neutrophils (10^3^/µ L)** | 3.40 (3.36-3.43) | 3.22 (3.19-3.25) | 3.35 (3.32-3.38) | 3.24 (3.21-3.27) | 3.20 (3.17-3.24) | -5.9 | 0.045 | |
| **Lymphocytes (10^3^/µ L)** | 1.71 (1.70-1.71) | 1.69 (1.68-1.69) | 1.71 (1.70-1.72) | 1.71 (1.70-1.72) | 1.68 (1.67-1.69) | -1.8 | 0.766 | |
| **Platelets (10^3^/µ L)** | 229 (227-230) | 227 (225-228) | 230 (229-232) | 228 (226-230) | 227 (226-229) | -0.7 | 0.290 | |
| **GDF-15 (pg/mL)** | 1265 (1240-1291) | 1228 (1203-1254) | 1239 (1213-1265) | 1247 (1221-1274) | 1262 (1235-1289) | -0.3 | 0.42 | |
| **IL-6 (pg/mL)** | 2.5 (2.5-2.6) | 2.5 (2.4-2.5) | 2.4 (2.4-2.5) | 2.4 (2.4-2.5) | 2.4 (2.4-2.5) | -0.1 | 0.139 | |
| **Renal function** | |  |  |  |  |  |  | |
| **Serum creatinine (mg/dL)** | 0.80 (0.79-0.81) | 0.77 (0.76-0.78) | 0.79 (0.78-0.80) | 0.79 (0.78-0.80) | 0.78 (0.78-0.79) | -2.5 | | 0.317 |
| **CKD-EPI (mL/min/1.73 m^2^)** | 83.0 (82.3-83.8) | 83.6 (82.9-84.4) | 83.6 (82.8-84.4) | 84.3 (83.5-85.1) | 83.7 (83.0-84.5) | 0.8 | | 0.213 |
| **Urinary albumin** | 7.53 (7.27-7.80) | 6.23 (6.06-6.42) | 6.75 (6.52-7.00) | 6.42 (6.20-6.65) | 6.60 (6.38-6.82) | -12.4 | | 0.155 |
| **Serum uric acid (mg/dL)** | 5.45 (5.38-5.53) | 5.30 (5.24-5.37) | 5.14 (5.07-5.21) | 5.18 (5.11-5.26) | 4.95 (4.88-5.02) | -9.2 | | <0.001 |
| **Blood sodium (mEq/L)** | 140.7 (140.7-140.8) | 140.6 (140.5-140.6) | 140.5 (140.5-140.6) | 140.5 (140.4-140.5) | 140.6 (140.6-140.7) | -0.1 | | 0.357 |
| **Blood potassium (mEq/L)** | 4.38 (4.37-4.40) | 4.46 (4.45-4.47) | 4.51 (4.50-4.53) | 4.49 (4.47-4.50) | 4.54 (4.53-4.56) | 3.7 | | 0.003 |
| **Hormones and nutritional biomarkers** | |  |  |  |  |  |  | |
| **TSH (μUI/mL)** | 1.87 (1.86-1.89) | 1.86 (1.58-1.61) | 1.56 (1.54-1.58) | 1.75 (7.73-1.77) | 1.66 (1.64-1.68) | -11.3 | 0.118 | |
| **Serum vitamin D (mg/dL)** | 16.7 (16.4-16.9) | 17.4 (17.2-17.6) | 17.2 (16.9-17.4) | 18.8 (18.5-19.1) | 17.9 (17.7-18.2) | 7.2 | 0.007 | |
| **Serum albumin (g/dL)** | 4.32 (4.32-4.32) | 4.31 (4.30-4.31) | 4.28 (4.27-4.28) | 4.29 (4.29-4.30) | 4.27 (4.27-4.27) | -1.2 | 0.005 | |
| **Total serum protein (g/dL)** | 7.04 (7.04-7.05) | 7.02 (7.01-7.02) | 7.03 (7.03-70.4) | 7.02 (7.02-7.03) | 7.06 (7.06-7.07) | 0.3 | 0.768 | |

hs-CRP: high sensitivity-C reactive protein; WBC: white blood cells; GDF-15: Growth differentiation factor 15; IL-6: Interleukin 6; CKD-EPI: Chronic Kidney Disease-Epidemiology Collaboration equation; TSH: Thyroid-stimulating hormone.

All analyses were adjusted for sex, age (continuous), energy intake (continuous), educational level (primary or less, secondary, university), smoking status (former, current, never), alcohol consumption (continuous), former-drinker status (yes, no), recreational physical activity in METs·hours/week (continuous), household physical activity in METs·hours/week (continuous), hours of television (continuous), number of chronic diseases (continuous), number of prescribed medications (continuous), very long chain omega-3 fatty acids (continuous), and fiber consumption (continuous).

^†^ *p* linear for trend of outcome variables when quintiles of consumption were used as a continuous variable.

**Supplementary Table S4.** Adjusted means (95% confidence interval), percentage difference, and *p* for linear trend across quintiles of roasting food consumption.

|  | **Roasting food consumption** | | | | |  | | |
| --- | --- | --- | --- | --- | --- | --- | --- | --- |
|  | **Q1** | **Q2** | **Q3** | **Q4** | **Q5** | **PD** | | ***p***^†^ |
| **Inflammatory markers** | |  |  |  |  |  |  | |
| **hs-CRP (mg/L)** | 1.16 (1.11-1.21) | 1.27 (122-1.32) | 1.17 (1.12-1.22) | 1.23 (1.18-1.29) | 1.10 (1.04-1.14) | -5.3 | 0.302 | |
| **WBC (10^3^/µ L)** | 5.76 (5.71-5.80) | 5.76 (5.72-5.80) | 5.76 (7.72-5.80) | 5.82 (5.78-5.87) | 5.72 (5.67-5.76) | -0.7 | 0.576 | |
| **Neutrophils (10^3^/µ L)** | 3.29 (2.26-3.33) | 3.29 (3.26-3.32) | 3.28 (2.25-3.31) | 3.30 (3.27-3.33) | 3.24 (3.20-3.27) | -1.5 | 0.181 | |
| **Lymphocytes (10^3^/µ L)** | 1.68 (1.67-1.69) | 1.69 (1.68-1.70) | 1.69 (1.68-1.69) | 1.75 (1.74-1.76) | 1.68 (1.67-1.69) | 0.0 | 0.483 | |
| **Platelets (10^3^/µ L)** | 228 (227-230) | 224 (223-226) | 227 (226-229) | 231 (229-232) | 231 (229.-232) | 1.2 | 0.087 | |
| **GDF-15 (pg/mL)** | 1231 (1205-1258) | 1272 (1245-1299) | 1220 (1196-1245) | 1255 (1229-1281) | 1263 (1237-1291) | 2.5 | 0.924 | |
| **IL-6 (pg/mL)** | 2.4 (2.3-2.4) | 2.5 (2.4-2.5) | 2.5 (2.5-2.6) | 2.3 (2.3-2.4) | 2.6 (2.5-2.6) | 7.7 | 0.800 | |
| **Renal function** | |  |  |  |  |  |  | |
| **Serum creatinine (mg/dL)** | 0.78 (0.77-0.79) | 0.79 (0.78-0.80) | 0.80 (0.79-0.81) | 0.77 (0.76-0.78) | 0.79 (0.78-0.80) | 0.9 | | 0.355 |
| **CKD-EPI (mL/min/1.73 m^2^)** | 83.5 (82.7-84.3) | 83.3 (82.5-84.1) | 83.8 (83.1-84.6) | 84.0 (83.3-84.7) | 83.6 (82.8-84.5) | 0.1 | | 0.134 |
| **Urinary albumin** | 6.72 (6.51-6.93) | 7.32 (7.08-7.56) | 7.21 (6.98-7.45) | 6.04 (5.85-6.24) | 6.10 (5.86-6.36) | -9.2 | | 0.034 |
| **Serum uric acid (mg/dL)** | 5.24 (5.18-5.31) | 5.35 (5.29-542) | 5.38 (5.30-5.46) | 5.20 (5.13-5.27) | 4.79 (4.72-4.86) | -8.6 | | 0.003 |
| **Blood sodium (mEq/L)** | 140.5 (140.5-140.6) | 140.6 (1406.-140.6) | 140.4 (140.4-140.5) | 140.8 (140.7-140.8) | 140.6 (140.5-140.6) | 0.1 | | 0.931 |
| **Blood potassium (mEq/L)** | 4.44 (4.43-4.45) | 4.44 (4.43-4.45) | 4.49 (4.47-4.50) | 4.49 (4.48-4.51) | 4.55 (4.54-4.57) | 2.5 | | 0.016 |
| **Hormones and nutritional biomarkers** | |  |  |  |  |  |  | |
| **TSH (μUI/mL)** | 1.58 (1.57-1.60) | 1.74 (1.72-1.75) | 1.76 (1.74-1.78) | 1.66 (1.65-1.68) | 1.71 (1.69-1.73) | 8.2 | 0.479 | |
| **Serum vitamin D (mg/dL)** | 18.3 (18.1-18.5) | 17.8 (17.6-18.0) | 16.80 (16.6-17.0) | 17.0 (16.8-17.2) | 17.8 (17.5-18.1) | -2.7 | 0.438 | |
| **Serum albumin (g/dL)** | 4.30 (4.29-4.30) | 4.32 (4.32-4.33) | 4.28 (4.27-4.28) | 4.28 (4.27-4.28) | 4.29 (4.28-4.29) | -0.2 | 0.267 | |
| **Total serum protein (g/dL)** | 7.06 (7.05-7.06) | 7.04 (7.04-7.05) | 7.04 (7.04-7.05) | 7.03 (7.02-7.03) | 7.02 (7.01-7.03) | -0.6 | 0.233 | |

hs-CRP: high sensitivity-C reactive protein; WBC: white blood cells; GDF-15: Growth differentiation factor 15; IL-6: Interleukin 6; CKD-EPI: Chronic Kidney Disease-Epidemiology Collaboration equation; TSH: Thyroid-stimulating hormone.

All analyses were adjusted for sex, age (continuous), energy intake (continuous), educational level (primary or less, secondary, university), smoking status (former, current, never), alcohol consumption (continuous), former-drinker status (yes, no), recreational physical activity in METs·hours/week (continuous), household physical activity in METs·hours/week (continuous), hours of television (continuous), number of chronic diseases (continuous), number of prescribed medications (continuous), very long chain omega-3 fatty acids (continuous), and fiber consumption (continuous).

^†^ *p* linear for trend of outcome variables when quintiles of consumption were used as a continuous variable.

**Supplementary Table S5.** Adjusted means (95% confidence interval), percentage difference, and *p* for linear trend across quintiles of pan-frying food consumption.

|  | **Pan-frying food consumption** | | | | |  | | |
| --- | --- | --- | --- | --- | --- | --- | --- | --- |
|  | **Q1** | **Q2** | **Q3** | **Q4** | **Q5** | **PD** | | ***P***^†^ |
| **Inflammatory markers** | |  |  |  |  |  |  | |
| **hs-CRP (mg/L)** | 1.38 (1.32-1.44) | 1.23 (1.18-1.28) | 1.22 (1.17-1.28) | 1.09 (1.04-1.13) | 1.06 (1.02-1.10) | -23.2 | 0.103 | |
| **WBC (10^3^/µ L)** | 5.86 (5.81-5.90) | 5.75 (5.71-5.79) | 5.69 (5.65-5.73) | 5.74 (5.70-5.79) | 5.78 (5.74-5.82) | -1.4 | 0.549 | |
| **Neutrophils (10^3^/µ L)** | 3.35 (3.32-3.39) | 3.27 (3.24-2.30) | 3.24 (3.21-3.27) | 3.29 (3.25-3.32) | 3.25 (3.22-3.29) | -3.0 | 0.831 | |
| **Lymphocytes (10^3^/µ L)** | 1.71 (1.70-1.72) | 1.71 (1.70-1.72) | 1.66 (1.65-1.66) | 1.68 (1.67-1.68) | 1.74 (1.73-1.75) | 1.75 | 0.363 | |
| **Platelets (10^3^/µ L)** | 230 (229-232) | 227 (225-228) | 231 (229-232) | 228 (227-230) | 225 (224-227) | -2.1 | 0.384 | |
| **GDF-15 (pg/mL)** | 1296 (1266-1327) | 1272 (1247-1297) | 1230 (1206-1255) | 1235 (1210-1261) | 1209 (1184-1234) | -4.9 | 0.742 | |
| **IL-6 (pg/mL)** | 2.6 (2.5-2.6) | 2.5 (2.5-2.6) | 2.4 (2.3-2.4) | 2.4 (2.4-2.5) | 2.3 (2.3-2.4) | -11.5 | 0.404 | |
| **Renal function** | |  |  |  |  |  |  | |
| **Serum creatinine (mg/dL)** | 0.80 (0.79-0.81) | 0.80 (0.78-0.80) | 0.79 (0.78-0.80) | 0.79 (0.78-0.80) | 0.80 (0.76-0.78) | -3.8 | | 0.201 |
| **CKD-EPI (mL/min/1.73 m^2^)** | 82.6 (81.8-83.4) | 83.6 (82.8-84.3) | 83.6 (82.9-84.4) | 84.1 (83.3-84.9) | 84.5 (83.7-85.2) | 2.3 | | 0.794 |
| **Urinary albumin** | 7.63 (0.32-7.94) | 6.56 (6.37-6.75) | 6.03 (5.84-6.22) | 7.02 (6.80-7.25) | 6.39 (6.20-6.59) | -16.3 | | 0.904 |
| **Serum uric acid (mg/dL)** | 5.20 (5.13-5.28) | 5.23 (5.16-5.30) | 5.38 (5.30-5.45) | 5.26 (5.19-5.33) | 4.98 (4.91-5.05) | -4.2 | | 0.204 |
| **Blood sodium (mEq/L)** | 140.6 (140.6-140.6) | 140.6 (140.5-140.6) | 140.5 (140.5-140.6) | 140.7 (140.7-104.7) | 140.6 (140.5-140.6) | 0.0 | | 0.951 |
| **Blood potassium (mEq/L)** | 4.49 (4.474.50) | 4.48 (4.47-4.49) | 4.49 (4.48-4.50) | 4.45 (4.44-4.46) | 4.47 (4.46-4.49) | -0.5 | | 0.316 |
| **Hormones and nutritional biomarkers** | |  |  |  |  |  |  | |
| **TSH (μUI/mL)** | 1.79 (1.77-1.81) | 1.61 (1.60-1.63) | 1.63 (1.61-1.64) | 1.75 (1.73-1.76) | 1.66 (1.64-1.67) | -7.3 | 0.473 | |
| **Serum vitamin D (mg/dL)** | 16.5 (16.3-16.8) | 16.7 (16.5-16.9) | 18.2 (18.0-18.4) | 18.2 (18.0-18.4) | 18.3 (18.1-18.6) | 10.9 | 0.033 | |
| **Serum albumin (g/dL)** | 4.29 (4.29-4.30) | 4.27 (4.27-4.28) | 4.30 (4.29-4.30) | 4.31 (4.31-4.32) | 4.29 (4.29-4.30) | 0.0 | 0.376 | |
| **Total serum protein (g/dL)** | 7.07 (7.06-7.08) | 7.02 (7.01-7.02) | 7.02 (7.01-7.02) | 7.03 (7.02-7.03) | 7.05 (7.05-7.06) | -0.3 | 0.938 | |

hs-CRP: high sensitivity-C reactive protein; WBC: white blood cells; GDF-15: Growth differentiation factor 15; IL-6: Interleukin 6; CKD-EPI: Chronic Kidney Disease-Epidemiology Collaboration equation; TSH: Thyroid-stimulating hormone.

All analyses were adjusted for sex, age (continuous), energy intake (continuous), educational level (primary or less, secondary, university), smoking status (former, current, never), alcohol consumption (continuous), former-drinker status (yes, no), recreational physical activity in METs·hours/week (continuous), household physical activity in METs·hours/week (continuous), hours of television (continuous), number of chronic diseases (continuous), number of prescribed medications (continuous), very long chain omega-3 fatty acids (continuous), and fiber consumption (continuous).

^†^ *p* linear for trend of outcome variables when quintiles of consumption were used as a continuous variable.

**Supplementary Table S6.** Adjusted means (95% confidence interval), percentage difference, and *p* for linear trend across quintiles of frying food consumption.

|  | **Frying food consumption** | | | | |  | | |
| --- | --- | --- | --- | --- | --- | --- | --- | --- |
|  | **Q1** | **Q2** | **Q3** | **Q4** | **Q5** | **PD** | | ***p***^†^ |
| **Inflammatory markers** | |  |  |  |  |  |  | |
| **hs-CRP (mg/L)** | 1.01 (0.98-1.05) | 1.16 (1.11-1.21) | 1.25 (1.20-1.30) | 1.35 (1.30-1.40) | 1.27 (1.21-1.33) | 25.7 | 0.217 | |
| **WBC (10^3^/µ L)** | 5.60 (5.56-5.65) | 5.79 (5.75-5.83) | 5.88 (5.84-5.82) | 5.78 (5.74-5.83) | 5.77 (5.72-5.80) | 3.0 | 0.406 | |
| **Neutrophils (10^3^/µ L)** | 3.15 (3.12-3.18) | 3.26 (3.23-3.30) | 3.38 (3.35-3.41) | 3.33 (3.30-3.36) | 3.28 (3.25-3.32) | 4.1 | 0.185 | |
| **Lymphocytes (10^3^/µ L)** | 1.69 (1.68-1.70) | 1.73 (1.72-1.74) | 1.69 (1.68-1.70) | 1.68 (1.67-1.69) | 1.71 (1.70-1.72) | 1.2 | 0.955 | |
| **Platelets (10^3^/µ L)** | 226 (225-228) | 230 (228-232) | 228 (226-229) | 227 (225-228) | 230 (229-232) | 1.7 | 0.488 | |
| **GDF-15 (pg/mL)** | 1219 (1191-1247) | 1215 (119-1241) | 1219 (1195-1244) | 1308 (1281-1335) | 1283 (1258-1309) | 5.0 | 0.008 | |
| **IL-6 (pg/mL)** | 2.4 (2.3-2.4) | 2.3 (2.3-2.4) | 2.5 (2.5-2.6) | 2.6 (2.5-2.6) | 2.5 (2.4-2.5) | 3.7 | 0.978 | |
| **Renal function** | |  |  |  |  |  |  | |
| **Serum creatinine (mg/dL)** | 0.78 (0.78-0.79) | 0.78 (0.77-0.79) | 0.78 (0.77-0.79) | 0.79 (0.78-0.80) | 0.79 (0.78-0.80) | 1.3 | | 0.317 |
| **CKD-EPI (mL/min/1.73 m^2^)** | 84.3 (83.5-85.1) | 84.3 (83.5-85.0) | 83.4 (82.6-84.2) | 83.5 (82.7-84.2) | 82.9 (82.1-83.6) | -1.7 | | 0.197 |
| **Urinary albumin** | 6.56 (6.35-6.78) | 6.44 (6.23-6.65) | 7.18 (6.95-7.42) | 6.70 (6.50-6.91) | 6.62 (6.37-6.88) | 0.9 | | 0.602 |
| **Serum uric acid (mg/dL)** | 5.23 (5.16-5.29) | 5.37 (5.30-5.44) | 5.09 (5.02-5.16) | 5.21(5.13-5.29) | 5.09 (5.01-5.17) | -2.7 | | 0.159 |
| **Blood sodium (mEq/L)** | 140.6 (140.6-140.7) | 140.5 (140.4-140.5) | 140.7 (140.6-140.7) | 140.5 (140.5-140.6) | 140.6 (140.6-140.6) | 0.0 | | 0.438 |
| **Blood potassium (mEq/L)** | 4.41 (4.39-4.42) | 4.47 (4.46-4.48) | 4.45 (1.43-4.46) | 4.52 (4.50-4.53) | 4.58 (4.56-4.60) | 3.9 | | 0.001 |
| **Hormones and nutritional biomarkers** | |  |  |  |  |  |  | |
| **TSH (μUI/mL)** | 1.61 (1.60-1.62) | 1.62 (1.60-1.63) | 1.72 (1.71-1.74) | 1.81 (1.80-1.83) | 1.70 (1.68-1.72) | 5.6 | 0.094 | |
| **Serum vitamin D (mg/dL)** | 19.1 (18.9-19.3) | 16.9 (16.7-17.1) | 17.9 (17.7-18.1) | 17.0 (16.7-17.2) | 16.7 (16.5-16.9) | -12.6 | 0.065 | |
| **Serum albumin (g/dL)** | 4.31 (4.30-4.31) | 4.28 (4.27-4.28) | 4.29 (4.28-4.29) | 4.29 (4.28-4.29) | 4.32 (4.31-4.32) | 0.2 | 0.286 | |
| **Total serum protein (g/dL)** | 7.05 (7.05-7.06) | 7.06 (7.06-7.07) | 7.04 (7.04-7.05) | 7.05 (7.04-7.05) | 7.00 (6.98-6.99) | -0.8 | 0.022 | |

hs-CRP: high sensitivity-C reactive protein; WBC: white blood cells; GDF-15: Growth differentiation factor 15; IL-6: Interleukin 6; CKD-EPI: Chronic Kidney Disease-Epidemiology Collaboration equation; TSH: Thyroid-stimulating hormone.

All analyses were adjusted for sex, age (continuous), energy intake (continuous), educational level (primary or less, secondary, university), smoking status (former, current, never), alcohol consumption (continuous), former-drinker status (yes, no), recreational physical activity in METs·hours/week (continuous), household physical activity in METs·hours/week (continuous), hours of television (continuous), number of chronic diseases (continuous), number of prescribed medications (continuous), very long chain omega-3 fatty acids (continuous), and fiber consumption (continuous).

^†^ *p* linear for trend of outcome variables when quintiles of consumption were used as a continuous variable.

**Supplementary Table S7.** Adjusted means (95% confidence interval), percentage difference, and p for linear trend across quintiles of toasting food consumption.

|  | **Toasting food consumption** | | | | |  | | |
| --- | --- | --- | --- | --- | --- | --- | --- | --- |
|  | **Q1** | **Q2** | **Q3** | **Q4** | **Q5** | **PD** | | ***p***^†^ |
| **Inflammatory markers** | |  |  |  |  |  |  | |
| **hs-CRP (mg/L)** | 1.31 (1.26-1.37) | 1.43 (1.36-1.51) | 1.34 (1.28-1.39) | 1.02 (0.985-1.06) | 1.03 (0.990-1.06) | -21.4 | 0.047 | |
| **WBC (10^3^/µ L)** | 5.96 (5.92-6.01) | 5.67 (5.62-5.72) | 5.72 (5.68-5.76) | 5.77 (5.73-5.81) | 5.62 (5.58-5.66) | -5.7 | 0.264 | |
| **Neutrophils (10^3^/µ L)** | 3.41 (3.38-3.44) | 3.26 (3.22-3.29) | 3.27 (3.24-3.31) | 3.28 (3.24-3.31) | 3.16 (3.13-3.19) | -7.3 | 0.253 | |
| **Lymphocytes (10^3^/µ L)** | 1.72 (1.72-1.73) | 1.65 (1.65-1.66) | 1.68 (1.68-1.69) | 1.72 (1.71-1.73) | 1.69 (1.68-1.70) | -2.3 | 0.985 | |
| **Platelets (10^3^/µ L)** | 229 (228-230) | 231 (229-233) | 228 (226-229) | 227 (226-229) | 227 (225-228) | -1.1 | 0.637 | |
| **GDF-15 (pg/mL)** | 1302 (1277-1327) | 1245 (1215-1276) | 1240 (1215-1266) | 1243 (1218-1269) | 1197 (1173-1222) | -8.0 | 0.857 | |
| **IL-6 (pg/mL)** | 2.7 (2.6-2.7) | 2.6 (2.6-2.7) | 2.4 (2.4-2.5) | 2.2 (2.2-2.3) | 2.4 (2.3-2.4) | -11.1 | 0.009 | |
| **Renal function** | |  |  |  |  |  |  | |
| **Serum creatinine (mg/dL)** | 0.80 (0.79-0.81) | 0.77 (0.76-0.78) | 0.79 (0.78-0.80) | 0.79 (0.78-0.80) | 0.78 (0.77-0.78) | -1.8 | | 0.782 |
| **CKD-EPI (mL/min/1.73 m^2^)** | 82.4 (81.8-83.1) | 85.5 (84.6-86.3) | 83.8 (83.0-84.6) | 83.9 (83.1-84.6) | 83.6 (82.8-84.4) | 1.5 | | 0.636 |
| **Urinary albumin** | 6.84 (6.60-7.09) | 6.96 (6.69-7.24) | 6.66 (6.44-6.88) | 6.58 (6.38-6.78) | 6.52 (6.33-6.73) | -4.7 | | 0.841 |
| **Serum uric acid (mg/dL)** | 5.26 (5.19-5.33) | 5.20 (5.11-5.29) | 5.28 (5.21-5.36) | 5.25(5.18-5.32) | 5.06 (4.99-5.13) | -3.8 | | 0.253 |
| **Blood sodium (mEq/L)** | 140.5 (140.5-140.6) | 140.5 (140.4-140.6) | 140.5 (140.5-140.6) | 140.6 (140.6-140.7) | 140.7 (140.6-140.7) | 0.1 | | 0.354 |
| **Blood potassium (mEq/L)** | 4.47 (4.46-4.79) | 4.45 (4.43-4.47) | 4.52 (4.50-4.53) | 4.52 (4.44-4.46) | 4.48 (4.47-4.49) | 0.2 | | 0.726 |
| **Hormones and nutritional biomarkers** | |  |  |  |  |  |  | |
| **TSH (μUI/mL)** | 1.68 (1.67-1.70) | 1.69 (1.67-1.70) | 1.71 (1.69-1.73) | 1.69 (1.68-1.71) | 1.65 (1.63-1.66) | -1.8 | 0.611 | |
| **Serum vitamin D (mg/dL)** | 17.0 (16.7-17.2) | 16.3 (16.0-16.5) | 17.2 (17.0-17.4) | 17.9 (17.8-18.2) | 18.8 (18.6-19.0) | 10.6 | 0.045 | |
| **Serum albumin (g/dL)** | 4.29 (4.28-4.29) | 4.29 (4.28-4.29) | 4.30 (4.30-4.31) | 4.31 (4.30-4.31) | 4.28 (4.28-4.29) | -0.2 | 0.964 | |
| **Total serum protein (g/dL)** | 7.03 (7.02-7.03) | 7.05 (7.04-7.06) | 7.04 (7.04-7.05) | 7.05 (7.04-7.06) | 7.02 (7.01-7.02) | -0.1 | 0.718 | |

hs-CRP: high sensitivity-C reactive protein; WBC: white blood cells; GDF-15: Growth differentiation factor 15; IL-6: Interleukin 6; CKD-EPI: Chronic Kidney Disease-Epidemiology Collaboration equation; TSH: Thyroid-stimulating hormone.

All analyses were adjusted for sex, age (continuous), energy intake (continuous), educational level (primary or less, secondary, university), smoking status (former, current, never), alcohol consumption (continuous), former-drinker status (yes, no), recreational physical activity in METs·hours/week (continuous), household physical activity in METs·hours/week (continuous), hours of television (continuous), number of chronic diseases (continuous), number of prescribed medications (continuous), very long chain omega-3 fatty acids (continuous), and fiber consumption (continuous).

^†^ *p* linear for trend of outcome variables when quintiles of consumption were used as a continuous variable.

**Supplementary Table S8.** Adjusted means (95% confidence interval), percentage difference, and *p* for linear trend across quintiles of sautéing food consumption.

|  | **Sautéing food consumption** | | | | |  | | |
| --- | --- | --- | --- | --- | --- | --- | --- | --- |
|  | **Q1** | **Q2** | **Q3** | **Q4** | **Q5** | **PD** | | ***p***^†^ |
| **Inflammatory markers** | |  |  |  |  |  |  | |
| **hs-CRP (mg/L)** | 1.06 (1.02-1.11) | 1.37 (1.32-1.43) | 1.25 (1.19-1.30) | 1.25 (1.20-1.30) | 1.05 (1.01-1.09) | -1.0 | 0.483 | |
| **WBC (10^3^/µ L)** | 5.70 (5.66-5.74) | 5.82 (5.77-5.86) | 5.76 (5.72-5.80) | 5.77 (5.73-5.81) | 5.77 (5.72-5.81) | 1.2 | 0.958 | |
| **Neutrophils (10^3^/µ L)** | 3.27 (3.24-3.30) | 3.33 (3.30-3.37) | 3.29 (3.25-3.32) | 3.25 (3.22-3.28) | 3.27 (2.23-3.30) | 0.0 | 0.316 | |
| **Lymphocytes (10^3^/µ L)** | 1.66 (1.66-1.67) | 1.68 (1.67-1.69) | 1.69 (1.69-1.70) | 1.74 (1.73-1.75) | 1.72 (1.71-1.72) | 3.6 | 0.079 | |
| **Platelets (10^3^/µ L)** | 226 (224-227) | 231 (229-232) | 230 (228-231) | 232 (230-233) | 223 (222-225) | -1.2 | 0.294 | |
| **GDF-15 (pg/mL)** | 1288 (1261-1316) | 1301 (1273-1330) | 1189 (1165-1214) | 1250 (1226-1276) | 1215 (1191-1234) | -5.7 | 0.041 | |
| **IL-6 (pg/mL)** | 2.4 (2.4-2.5) | 2.5 (2.5-2.6) | 2.5 (2.5-2.6) | 2.4 (2.4-2.5) | 2.4 (2.4-2.5) | 0.0 | 0.851 | |
| **Renal function** | |  |  |  |  |  |  | |
| **Serum creatinine (mg/dL)** | 0.81 (0.80-0.81) | 0.80 (0.79-0.81) | 0.77 (0.76-0.78) | 0.78 (0.77-0.79) | 0.78 (0.77-0.79) | -3.4 | | 0.004 |
| **CKD-EPI (mL/min/1.73 m^2^)** | 82.5 (81.7-83.3) | 82.8 (82.0-83.6) | 84.1 (83.4-84.9) | 84.3 (83.5-85.1) | 84.6 (83.9-85.4) | 2.6 | | 0.020 |
| **Urinary albumin** | 7.13 (6.90-7.36) | 6.51 (6.28-6.74) | 6.44 (6.22-6.67) | 6.53 (6.32-6.74) | 6.80 (6.57-7.04) | -4.6 | | 0.376 |
| **Serum uric acid (mg/dL)** | 5.31 (5.24-5.38) | 5.27 (5.20-5.34) | 5.22 (5.15-5.30) | 5.17 (5.10-5.24) | 5.06 (4.99-5.13) | -4.7 | | 0.003 |
| **Blood sodium (mEq/L)** | 140.6 (140.5-140.6) | 140.3 (140.3-140.4) | 140.5 (140.4-140.5) | 140.8 (140.7-140.8) | 140.7 (140.7-140.8) | 0.1 | | 0.074 |
| **Blood potassium (mEq/L)** | 4.44 (4.42-4.45) | 4.59 (4.58-4.60) | 4.46 (4.45-4.47) | 4.49 (4.48-4.50) | 4.40 (4.39-4.42) | -1.0 | | 0.239 |
| **Hormones and nutritional biomarkers** | |  |  |  |  |  |  | |
| **TSH (μUI/mL)** | 1.64 (1.63-1.66) | 1.77 (1.75-1.79) | 1.71 (1.69-1.72) | 1.62 (1.60-1.63) | 1.70 (1.68-1.72) | 3.7 | 0.982 | |
| **Serum vitamin D (mg/dL)** | 17.4 (17.2-17.6) | 16.7 (16.5-16.9) | 18.3 (18.1-18.5) | 17.6 (17.3-17.8) | 18.0 (17.5-18.3) | 3.5 | 0.202 | |
| **Serum albumin (g/dL)** | 4.30 (4.29-4.30) | 4.28 (4.28-4.29) | 4.28 (4.27-4.28) | 4.30 (4.30-4.31) | 4.30 (42.9-4.31) | 0.0 | 0.485 | |
| **Total serum protein (g/dL)** | 7.04 (7.0-7.04) | 7.01 (7.01-7.02) | 7.00 (6.99-7.00) | 7.09 (7.09-7.10) | 7.05 (7.04-7.05) | 0.1 | 0.121 | |

hs-CRP: high sensitivity-C reactive protein; WBC: white blood cells; GDF-15: Growth differentiation factor 15; IL-6: Interleukin 6; CKD-EPI: Chronic Kidney Disease-Epidemiology Collaboration equation; TSH: Thyroid-stimulating hormone.

All analyses were adjusted for sex, age (continuous), energy intake (continuous), educational level (primary or less, secondary, university), smoking status (former, current, never), alcohol consumption (continuous), former-drinker status (yes, no), recreational physical activity in METs·hours/week (continuous), household physical activity in METs·hours/week (continuous), hours of television (continuous), number of chronic diseases (continuous), number of prescribed medications (continuous), very long chain omega-3 fatty acids (continuous), and fiber consumption (continuous).

^†^ *p* linear for trend of outcome variables when quintiles of consumption were used as a continuous variable.

**Supplementary Table S9.** Adjusted means (95% confidence interval), percentage difference, and *p* for linear trend across quintiles of stewing food consumption.

|  | **Stewing food consumption** | | | | |  | | |
| --- | --- | --- | --- | --- | --- | --- | --- | --- |
|  | **Q1** | **Q2** | **Q3** | **Q4** | **Q5** | **PD** | | ***p***^†^ |
| **Inflammatory markers** | |  |  |  |  |  |  | |
| **hs-CRP (mg/L)** | 1.13 (1.08-1.18) | 1.20 (1.15-1.25) | 1.16 (1.11-1.21) | 1.19 (1.14-1.24) | 1.28 (1.23-1.34) | 13.3 | 0.817 | |
| **WBC (10^3^/µ L)** | 5.87 (5.83-5.92) | 5.81 (5.76-5.85) | 5.72 (5.68-5.76) | 5.74 (5.70-5.79) | 5.68 (5.64-5.72) | -3.2 | 0.034 | |
| **Neutrophils (10^3^/µ L)** | 3.30 (3.27-3.33) | 3.33(3.29-3.36) | 3.26 (2.23-3.30) | 3.28 (3.25-3.32) | 3.23 (3.20-3.26) | -2.1 | 0.273 | |
| **Lymphocytes (10^3^/µ L)** | 1.77 (1.76-1.78) | 1.68 (1.68-1.69) | 1.68 (1.67-1.68) | 1.67 (1.66-1.68) | 1.69 (1.68-1.70) | -4.5 | 0.021 | |
| **Platelets (10^3^/µ L)** | 229 (228-231) | 230 (229-232) | 226 (224-227) | 226 (224-227) | 230 (229-233) | 0.3 | 0.788 | |
| **GDF-15 (pg/mL)** | 1265 (1238-1293) | 1272 (1246-1299) | 1255 (1229-1281) | 1239 (1212-1266) | 1210 (1187-1234) | -4.3 | 0.688 | |
| **IL-6 (pg/mL)** | 2.4 (2.3-2.4) | 2.5 (2.5-2.6) | 2.5 (2.5-2.6) | 2.4 (2.4-2.4) | 2.5 (2.4-2.5) | 4.0 | 0.777 | |
| **Renal function** | |  |  |  |  |  |  | |
| **Serum creatinine (mg/dL)** | 0.79 (0.78-0.80) | 0.78 (0.77-0.78) | 0.79 (0.78-0.80) | 0.79 (0.78-0.80) | 0.78 (0.77-079) | -1.3 | | 0.976 |
| **CKD-EPI (mL/min/1.73 m^2^)** | 83.1 (82.3-83.9) | 83.2 (82.4-83.9) | 83.5 (82.7-84.2) | 84.1 (83.3-84.9) | 84.5 (83.8-85.3) | 1.7 | | 0.243 |
| **Urinary albumin** | 6.33 (6.10-5.57) | 7.06 (6.81-7.33) | 6.89 (6.68-7.11) | 6.82 (6.62-7.04) | 6.33 (6.14-6.54) | 0.0 | | 0.880 |
| **Serum uric acid (mg/dL)** | 5.18 (5.11-5.25) | 5.12 (5.05-5.19) | 5.14 (5.06-5.21) | 5.37 (5.30-5.45) | 5.23 (5.16-5.30) | 1.0 | | 0.035 |
| **Blood sodium (mEq/L)** | 140.6 (140.6-140.7) | 140.4 (140.4-140.5) | 140.5 (140.4-140.5) | 140.8 (140.8-140.8) | 140.6 (140.5-140.6) | 0.0 | | 0.558 |
| **Blood potassium (mEq/L)** | 4.45 (4.43-4.46) | 4.50 (4.48-4.51) | 4.50 (4.49-4.52) | 4.46 (4.45-4.48) | 4.47 (4.49-4.49) | 0.5 | | 0.715 |
| **Hormones and nutritional biomarkers** | |  |  |  |  |  |  | |
| **TSH (μUI/mL)** | 1.73 (1.71-1.75) | 1.73 (1.71-1.75) | 1.65 (1.63-1.67) | 1.64 (1.62-1.65) | 1.67 (1.66-1.69) | -3.5 | 0.320 | |
| **Serum vitamin D (mg/dL)** | 17.8 (17.5-18.0) | 16.9 (16.7-17.1) | 17.6 (17.3-17.8) | 17.1 (16.9-17.3) | 18.6 (18.3-18.8) | 4.5 | 0.252 | |
| **Serum albumin (g/dL)** | 4.31 (4.30-4.31) | 4.29 (4.28-4.29) | 4.28 (4.27-4.28) | 4.30 (4.29-4.30) | 4.29 (4.29-4.30) | -0.5 | 0.937 | |
| **Total serum protein (g/dL)** | 7.04 (7.04-7.05) | 7.03 (7.03-7.04) | 7.02 (7.01-7.02) | 7.04 (7.04-7.05) | 7.05 (7.05-7.06) | 0.1 | 0.690 | |

hs-CRP: high sensitivity-C reactive protein; WBC: white blood cells; GDF-15: Growth differentiation factor 15; IL-6: Interleukin 6; CKD-EPI: Chronic Kidney Disease-Epidemiology Collaboration equation; TSH: Thyroid-stimulating hormone.

All analyses were adjusted for sex, age (continuous), energy intake (continuous), educational level (primary or less, secondary, university), smoking status (former, current, never), alcohol consumption (continuous), former-drinker status (yes, no), recreational physical activity in METs·hours/week (continuous), household physical activity in METs·hours/week (continuous), hours of television (continuous), number of chronic diseases (continuous), number of prescribed medications (continuous), very long chain omega-3 fatty acids (continuous), and fiber consumption (continuous).

^†^ *p* linear for trend of outcome variables when quintiles of consumption were used as a continuous variable.
